# Comparing Lung Cancer Risks in Sweden, USA, and Japan

**DOI:** 10.5402/2012/687298

**Published:** 2012-03-07

**Authors:** Örjan Hallberg, Olle Johansson

**Affiliations:** ^1^Hallberg Independent Research, Brattforsgatan 3, 123 50 Farsta, Sweden; ^2^The Experimental Dermatology Unit, Department of Neuroscience, Karolinska Institute, 171 77 Stockholm, Sweden

## Abstract

*Objective*. To develop a conceptual model for lung cancer rates to describe and quantify observed differences between Sweden and USA contra Japan. *Method*. A two-parameter lognormal distribution was used to describe the lung cancer rates over time after a 1-year period of smoking. Based on that risk function in combination with smoking prevalence, the calculated age-standardized rates were adjusted to fit reported data from Japan, Sweden, and the USA by parameter variation. *Results*. The risk of lung cancer is less in Japan than in Sweden and in the USA at the same smoking prevalence and intensity. Calculated age-specific rates did also fit well to reported rates without further parameter adjustments. *Conclusions*. This new type of cancer model appears to have high degree of predictive value. It is recommended that data from more countries are studied to identify important life-style factors related to lung cancer.

## 1. Introduction

Prior to 1955, the relationship between smoking tobacco and lung cancer was not recognised as it is today. A 1955 Swedish encyclopaedia describes nervous problems, stomach problems, and throat problems, but not lung cancer, as possible results of heavy smoking [1]. However, after 1955, lung cancer mortality began to suddenly increase in Sweden, and older people in particular were subjected to a fast increasing risk of lung cancer.

The aim of this study is to develop a conceptual model for predicting lung cancer rates. Such means may be useful in promoting successful preventive work against lung cancer. In cancer epidemiology, statistical models use linear and logistic regression to evaluate relationships between risk factors and cancer incidence. Less frequently used are biomathematical models translating a hypothesis about the biological process into mathematical terms [2].

There are differences between countries worldwide regarding lung cancer even if they have similar cigarette consumption per inhabitant and year [3]. This indicates that there are other causative factors, as well as different genetics, still not considered or yet identified in epidemiological studies of lung cancer.

The model developed for this study was based on a similar model, successfully used for estimating melanoma rates over time, taking reduced repair efficiency of sun-induced skin damages into consideration [4]. In the current study, the model developed for lung cancer had to account for varying smoking prevalence over time. 

The main finding from this study is that the model with the parameters used was capable of predicting age-specific incidence for both men and women in Sweden and for men in the USA and Japan. The model study indicates that the lung cancer risk after one year of smoking is larger in Sweden and USA as compared to Japan. This conclusion was strengthened by the fact that western countries in general have twice the lung cancer rate as nonwestern countries at equal cigarette consumption per person and year. A study of associations between lung cancer rates and life-style factors other than smoking is thus recommended.

## 2. Materials and Methods

Data on smoking prevalence were collected for men and women from official sources [5]. Age-specific smoking prevalence data have been reported in Sweden since 1980 and the total smoking prevalence since 1916. Smoking prevalence data from the USA and Japan were retrieved from [6, 7]. The expected life duration for smokers and non-smokers in western countries were obtained from survival plots given by Doll et al. [8].

This study sought to define a one-year period of average smoking for men in Sweden as the basic damage-generating unit causing a lung cancer risk that would increase by time according to a statistical function. The smoking intensity per lung volume among Swedish male smokers was defined as the norm in this model. The smoking intensities for men in Japan and the USA and for women in Sweden were adjusted to best fit data and then compared with available reported data. The probability function was used to calculate cancer cases from each smoking period of one year over the life time of a birth cohort consisting of 100,000 men or women. The cancer cases from all these smoking periods were then summed over time (calendar years) in a life matrix (see Table 1) to give the primary calculated incidence. This primary incidence versus age was finally adjusted for Sweden and the USA to account for the reduced fraction of smokers alive at old age due to the longer life expectancy of nonsmokers in western countries [8].

This analysis was repeated for all birth cohorts to calculate the age-standardized incidence over time. The statistical functions used were characterized by two statistical parameters: the dispersion (in time, decades) and time to reach 0.1% (used to define the median value) of a lognormal distribution. The parameters were first varied to identify the best fit of calculated age-standardized rates to reported rates in Japan. To verify the usefulness of the model, calculated age-specific incidences were compared with reported data without further parameter variation. The dispersion giving best fit to reported rates in Japan was also used for Sweden and the USA, while the time to 0.1% was further fine-tuned to fit the Swedish and US data. We considered the dispersion mainly to be a characteristic of the disease (lung cancer) so that it should be used for all countries. The time to 0.1% risk of cancer is more likely to be a characteristic of the environment and should thus be used as an adjustable parameter to fit data.

## 3. Results

**Figure 1:**
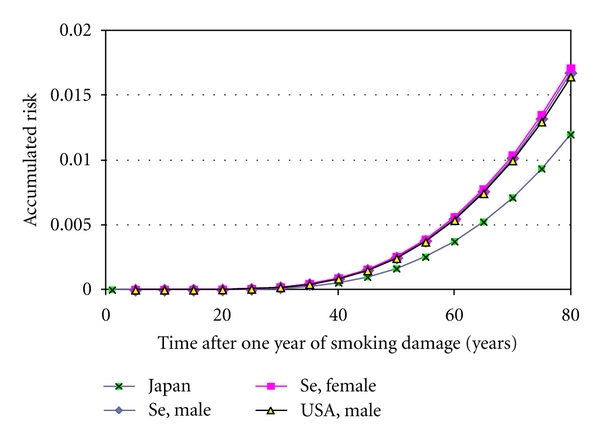
Accumulated lung cancer risk in Sweden and the USA and in Japan from one year of smoking.

**Figure 2:**
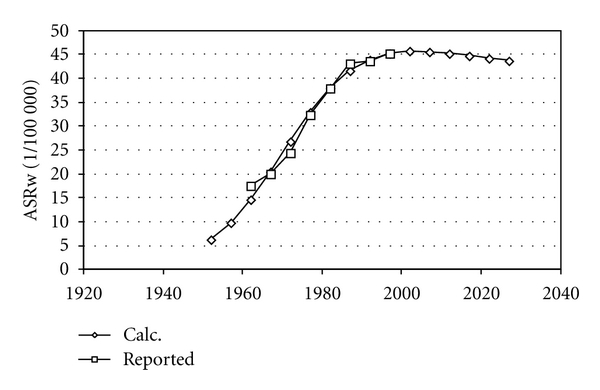
Calculated and reported age-standardized incidence for men in Japan.

**Figure 3:**
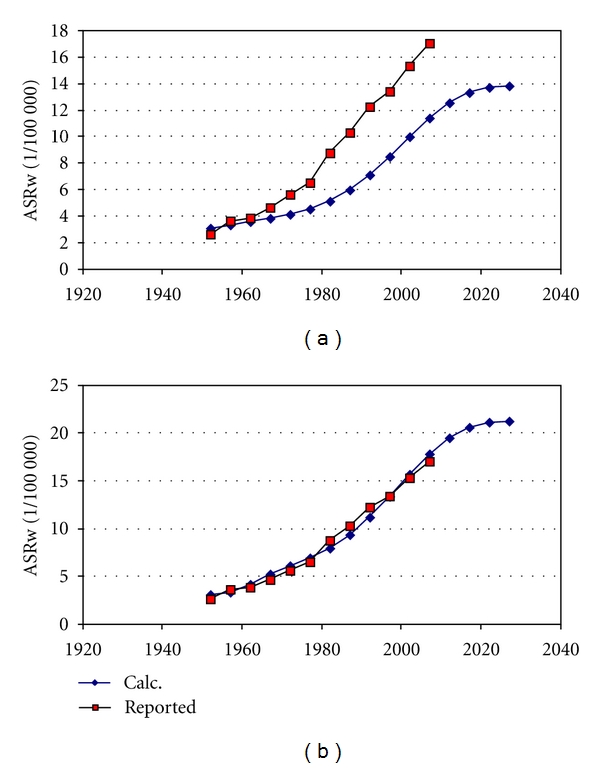
(a) Calculated and reported age-standardized incidence for women in Sweden using the same two parameters as were used for Japan in Figure 2. (b) Calculated and reported age-standardized incidence for women in Sweden after optimization of the parameter value for time to 0.1%.

Figure 1 shows the basic lung cancer risks in Sweden and the USA and in Japan from one year of smoking.  Figure 2 shows age-standardized rates for men in Japan after varying the two parameters (dispersion and time to 0.1%) to best fit calculated and reported data.  Figure 3(a) shows that the calculated incidence for Swedish women would have been about twice as low as actually reported. In order to better fit reported data, the time to 0.1% had to be adjusted somewhat which is shown in Figure 3(b) and in Table 2. The same dispersion as for Japan was used also for men in Sweden and in the USA and for women in Sweden.

**Figure 4:**
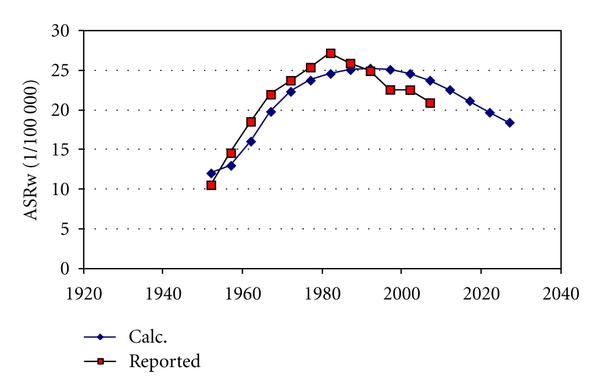
Calculated and reported age-standardized incidence for men in Sweden after optimization of the parameter value for time to 0.1%.

**Figure 5:**
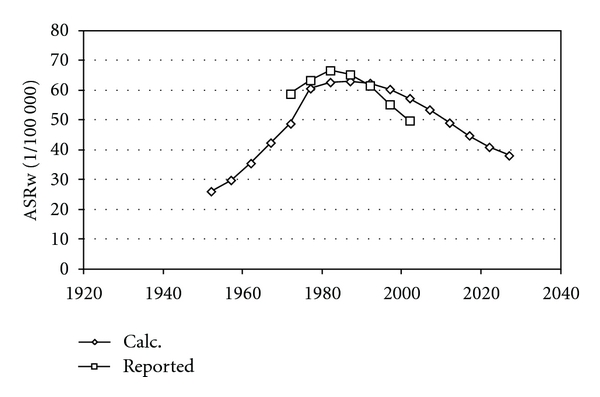
Calculated and reported age-standardized incidence for men in the USA after optimization of the parameter value for time to 0.1%.

**Figure 6:**
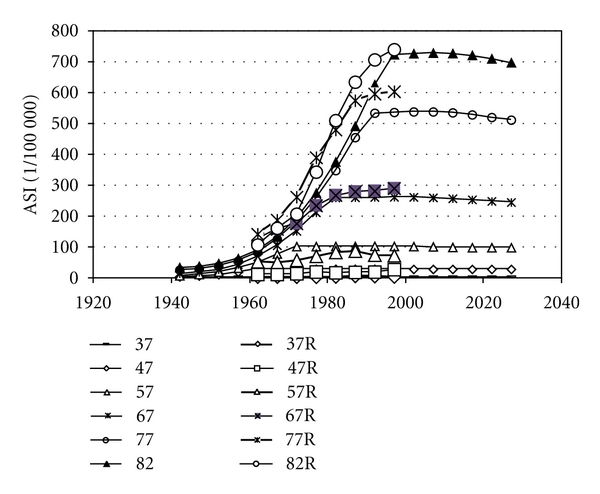
Calculated and reported age-specific lung cancer incidence for men over time in Japan. Data are given for age groups from 37 to 82 years, where *R* stands for reported data.

**Figure 7:**
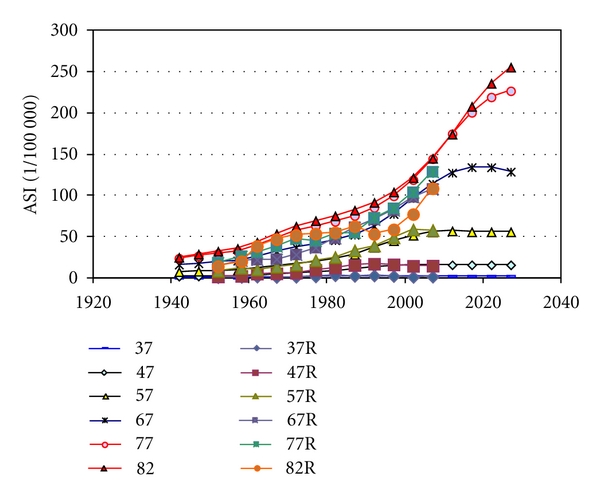
Calculated and reported age-specific lung cancer incidence for women over time in Sweden.

**Figure 8:**
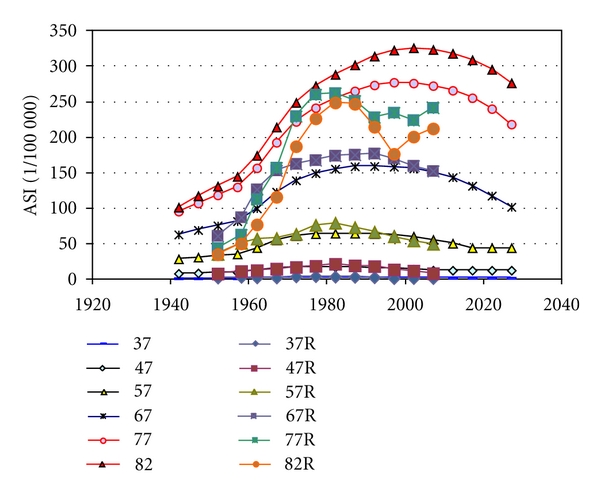
Calculated and reported age-specific lung cancer incidence for men over time in Sweden.

**Figure 9:**
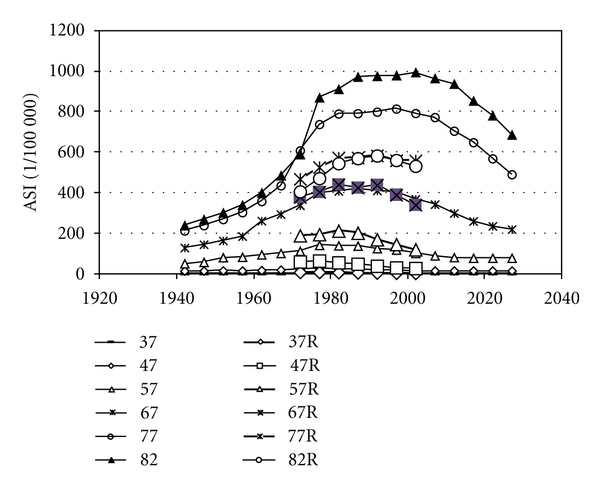
Calculated and reported age-specific lung cancer incidence for men over time in the USA.

We found the parameter values extracted from the statistics on women very similar to parameters to fit data on men in Sweden and in the USA (Figures 4 and 5). Table 2 gives the parameters for best fit to reported age-standardised rates, used to calculate all incidences in Figures 2–9.  Figure 6 shows reported and calculated age-specific lung cancer incidence for men in Japan. Similar results were obtained for men in Sweden and for men in the USA (Figures 8 and 9). It is of interest to note that the rates for men and women older than 80 years since around 1995 have been increasing (Figures 7 and 8). The sharp increase after 1997 for corresponding older men in Sweden cannot be explained by the model, however.

**Figure 10:**
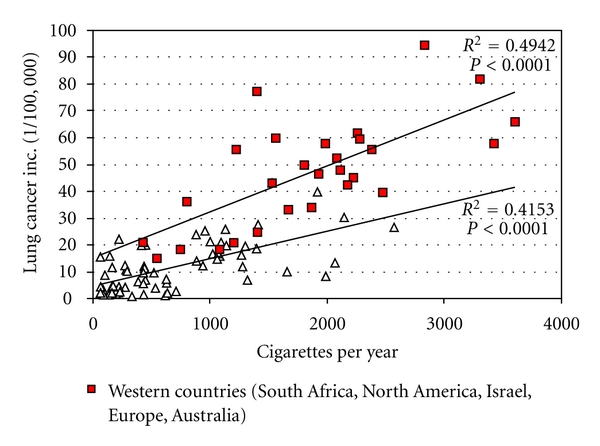
Lung cancer incidence versus cigarette consumption in western and nonwestern countries.

A study of data from international cancer registries [9] in combination with reported cigarette consumption per capita [10] showed that western countries have twice the amount of lung cancer compared to nonwestern countries at similar smoking levels (See Figure 10).

## 4. Discussion

We have here been using a mechanistic model requiring just two parameters instead of the empirical methods normally used, for example, in age-period cohort (APC) models [11, 12]. The rate (*R*) at any age (*a*) and period (*p*) can be calculated as

(1) $$\begin{array}{c} R_{ap}=\exp\left(A_{a}\;+\;D*p+P_{p}+C_{c}\right), \end{array}$$

Here *A*_*a*_ is a constant that varies by age, *D* is a constant that is multiplied by the period *p*, *P*_*p*_ is a specific constant added for each period *p*, and *C* is a specific constant for each cohort, for example, birth cohort.

In order to generate the age-specific incidence plots and age-standardised plots in Figures 2–9 using the APC model, quite a large number of parameters is necessary. Such a model of lung cancer mortality in relation to age, duration of smoking, and daily cigarette consumption was studied by Flanders et al. [13].

The connection between tobacco smoking and lung cancer has long been known (review in [14]). A comprehensive report on tobacco use and cancer was given in [6]. In order to take different smoking intensity patterns, defined as number of cigarettes per lung volume, between Sweden and USA into consideration, we allowed an intensity factor to be varied. Best fit was found when the smoking intensity was 3.3 times larger in the USA as compared to Sweden. The average annual weight of smoked tobacco per inhabitant during the period from 1922 to 2002 was 1.047 kg in Sweden and 3.318 kg in the USA or 3.2 times the Swedish weight. The computer also found a best fit for Swedish women using a smoking intensity of 1.19. Initially this was intriguing since Swedish smoking women tend to smoke 12 cigarettes per day compared with 15 for male smokers. However, taking their different lung volumes into consideration (4.2 vs. 6 litres), it turned out that 12/4.2∗6/15 = 1.14, a value which was then used for the calculations (Table 3).

We used information on life expectancy of smokers and nonsmokers in western countries to correct the model for decreasing ratios of smokers versus age among older people in Sweden and in the USA. The generally observed decline in cancer incidence for the oldest people was reviewed in [15]. Due to natural death, there will be an accumulation of survivors who have lived in good and healthy conditions and this will manifest itself in lower cancer rates. This also makes an argument to search for more specific information about what these good conditions really are.

The question why the overall cancer risk is higher in more developed countries was raised in [16]. One possible explanation was that the vulnerability has increased because the “immunity is affected by factors associated with economic progress and a western life-style.” Such factors may include diet, chemicals, or continuous exposure to immune-disturbing electromagnetic fields. Figure 10 shows lung cancer rates versus smoking habits in western and nonwestern countries.

Several studies report that there is a significant reduction in DNA repair capacity (DRC) found in lung cancer patients compared with healthy controls, [17, 18]. There are rapid methods for determination of DRC by blood tests to find candidates for intensive follow-up and possibly screening, [19]. By using such methods, it is also possible not only to compare lung cancer patients with healthy controls, but also to compare DRC among healthy smokers from different populations, for example, from Japan and the USA. One step in that direction was recently presented by Gandara et al. [20].

Our findings from this model study indicate a significant difference between the capability of coping with heavy smoking without getting lung cancer in Japan versus the USA, and Sweden. If DRC generally is better in Japan than in for example, the USA, then it is our firm belief that we immediately should study what environmental factors that might have the strongest influence on DRC and then use this information for better cancer prevention strategies.

Three major conclusions can be drawn from this study.

1. The aim of this study was to develop a conceptual model for predicting lung cancer rates. Such a model was indeed achieved and it will be an important task to follow it over time as well as applying it to statistical material from other countries.
2. A study of associations between lung cancer rates, DRC, and environmental and life-style factors other than smoking is strongly recommended.
3. Lung cancer rates in Sweden and the USA might become significantly reduced if we could identify and adapt to such relevant factors in Japan.

## Figures and Tables

**Table 1:** A life matrix based on annual smoking damage (*N*) and cancer risk (*F*) to get the accumulated risk over life time (vertical sum).

		Year							
Year	Dmg	*Y*_1_	*Y*_2_	*Y*_3_	*Y*_4_	*Y*_5_	*Y*_6_	*Y*_7_	⋯
*Y*_1_	*N*_1_	*N*_1_ *F*_1_	*N*_1_ *F*_2_	*N*_1_ *F*_3_	*N*_1_ *F*_4_	*N*_1_ *F*_5_	*N*_1_ *F*_6_	*N*_1_ *F*_7_	⋯
*Y*_2_	*N*_2_		*N*_2_ *F*_1_	*N*_2_ *F*_2_	*N*_2_ *F*_3_	*N*_2_ *F*_4_	*N*_2_ *F*_5_	*N*_2_ *F*_6_	⋯
*Y*_3_	*N*_3_			*N*_3_ *F*_1_	*N*_3_ *F*_2_	*N*_3_ *F*_3_	*N*_3_ *F*_4_	*N*_3_ *F*_5_	⋯
*Y*_4_	*N*_4_				*N*_4_ *F*_1_	*N*_4_ *F*_2_	*N*_4_ *F*_3_	*N*_4_ *F*_4_	⋯
*Y*_5_	*N*_5_					*N*_5_ *F*_1_	*N*_5_ *F*_2_	*N*_5_ *F*_3_	⋯
*Y*_6_	*N*_6_						*N*_6_ *F*_1_	*N*_6_ *F*_2_	⋯
*Y*_7_	*N*_7_							*N*_7_ *F*_1_	⋯

⋮	⋮	∑*Y*_1_	∑*Y*_2_	∑*Y*_3_	∑*Y*_4_	∑*Y*_5_	∑*Y*_6_	∑*Y*_7_	⋯

**Table 2:** Log-normal parameters for lung cancer risk functions.

Population	Dispersion (decades)	Time to 0.1% (Years)
Japan male	0.30	45.13
Se male, Se female USA male	0.30; 0.30 0.30	41.22; 41.00 41.43

**Table 3:** Smoking intensity relative to that of Swedish men.

Country, gender	Smoking intensity relative to Swedish men
Sweden, men	1
Sweden, women	1, 14
Japan, men	1, 24
USA, men	3, 30
